# Soil bacterial communities are influenced by soil chemical characteristics and dispersal limitation in commercial strawberry production systems

**DOI:** 10.1002/pei3.10099

**Published:** 2023-01-11

**Authors:** Nicholas LeBlanc, Samantha Gebben

**Affiliations:** ^1^ United States Department of Agriculture, Agricultural Research Service, Crop Improvement and Protection Research Unit Salinas California USA

**Keywords:** agricultural microbiomes, community assembly, soil health

## Abstract

Bacterial communities play multiple functional roles in soil that have positive and negative feedbacks on plant health. However, relatively few studies have focused on the ecology of soil bacterial communities in commercial strawberry production systems. The objective of this study was to determine if ecological processes influencing soil bacterial communities are consistent among commercial strawberry production locations and plots within the same geographic region. Soil samples were collected using a spatially explicit design from three plots in two commercial strawberry production locations in the Salinas Valley region of California. Soil carbon, nitrogen, and pH were measured for each of the 72 soil samples and bacterial communities were characterized using 16 S rRNA sequencing. Multivariate analyses showed bacterial community composition was differentiated between the two strawberry production locations. Analyses of communities within plots demonstrated soil pH and nitrogen were significant predictors of bacterial community composition in one of the three sampled plots. Bacterial communities displayed spatial structure in two plots at one location based on a significant increase in community dissimilarity with increasing spatial distance. Null model analyses identified a lack of phylogenetic turnover among bacterial communities in all plots, but a greater frequency of dispersal limitation in the two plots where spatial structure was also observed. Overall, this work suggests that ecological factors influencing soil bacterial communities are not consistent among different strawberry production locations or plots which may impact the ability to predict or manage the effect of soil microbiomes on strawberry health.

## Graphical abstract

Soil bacterial communities play important functional roles that feedback on crop health. However, factors influencing their composition and diversity in strawberry production systems are poorly understood. This study found the influence of soil chemical characteristics and dispersal limitation on soil bacterial communities in highly uniform strawberry production systems was not consistent among different locations and plots within the same geographic region.

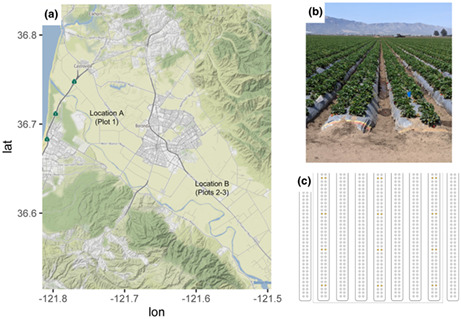

## 1 INTRODUCTION

Bacterial communities in soil have positive and negative feedbacks on crop health in agricultural ecosystems. In addition to their contribution to transformation of primary elements like nitrogen, bacteria can increase or decrease availability of other nutrients for plant uptake through mineralization and immobilization (Bodelier, 2011; Crowther et al., 2019; Falkowski et al., 2008; Jacoby et al., 2017; Kertezs & Mirleau, 2004). Multiple bacterial taxa have been shown to reduce the negative effects of yield limiting plant diseases by suppressing soilborne plant pathogens through production of antimicrobial secondary metabolites and competition for growth‐limiting resources (Schlatter et al., 2017; Weller et al., 2002). Soil is also a reservoir for bacteria that directly interact with plant roots as pathogens and mediators of biotic and abiotic stress tolerance (Berendsen et al., 2012; Pieterse et al., 2014; Trivedi et al., 2020; van Bruggen et al., 2015). Due to these variable impacts of bacteria, understanding factors that influence bacterial communities in soil is an essential precursor to managing their activity to improve crop health.

Factors influencing bacterial communities in soil can broadly be divided between those that play a role in deterministic (i.e., niche based) versus stochastic processes (Zhou & Ning, 2017). For example, multiple studies have shown variation in soil chemical characteristics such as pH are primary predictors of bacterial community composition in soil (Fierer, 2017; Lauber et al., 2008; Rousk et al., 2010; Shi et al., 2018; Yashiro et al., 2016). This relationship has been attributed to variation in pH tolerance (i.e., pH niche width) of different bacterial taxa as well as indirect effects of soil pH on other soil chemical characteristics that constrain bacterial growth (Fernández‐Calviño et al., 2011; Lammel et al., 2018). The composition of soil bacterial communities can also be influenced by stochastic processes that are not related to niche differences of bacterial taxa. The composition of bacterial communities in soil often increase in dissimilarity with increasing spatial distance among samples (Clark et al., 2021). This pattern is often interpreted as evidence of dispersal limitation (Hanson et al., 2012). Together, these examples highlight the importance of understanding selective factors related to soil chemical characteristics as well as the role that stochastic processes, like dispersal, play in structuring bacterial communities in soil.

Multiple theoretical frameworks and analytical approaches have been developed to quantify the importance of different deterministic and stochastic processes influencing microbial communities (Zhou & Ning, 2017). One approach that takes advantage of sequence‐based characterization of microbial communities is to measure the distribution of phylogenetic and taxonomic turnover among communities within a local environment (Stegen et al., 2012). Based on the assumption or demonstration that phylogenetic similarity is correlated with niche differences among microbial taxa, estimated metrics of phylogenetic turnover among samples can be compared to a null distribution to infer the frequency of underlying processes influencing microbial communities (Dini‐Andreote et al., 2015; Stegen et al., 2013; Stegen et al., 2015). For example, reduced phylogenetic turnover among samples in a local environment is attributed to homogenous selective pressures, while increased phylogenetic turnover is due to variable selective pressures. This null modeling approach has been widely applied to understand community assembly processes in aquatic and terrestrial ecosystems (Zhou & Ning, 2017). More recent use of this approach to study agricultural soils has shown that both deterministic and stochastic processes influence bacterial community composition in soil of these highly managed ecosystems (Barnett et al., 2019; Jiao & Lu, 2020; Li et al., 2021; Shi et al., 2018).

Commercial strawberry production in the United States is valued at more than $3.4 billion a year with most of production located on the West Coast in California (Samtani et al., 2019; USDA National Agricultural Statistics Service, 2017). Continued strawberry production in the United States and California is facing significant challenges. A primary challenge is the increasing regulation of soil fumigants used for pest management due to their negative impacts on the environment and human health (Fang et al., 2018; Guthman & Brown, 2016; Holmes et al., 2020; Pesonen & Vähäkangas, 2020). Recognition that soil fumigation is not a long‐term solution for strawberry production has motivated evaluation of alternative strategies for improving strawberry production. Among these alternative strategies is a growing interest in using beneficial microorganisms and managing soil microbiomes to improve strawberry health (Holmes et al., 2020; Martin, 2003; Mazzola et al., 2018). Recent research on soil microbiomes in strawberry production systems has shown soil bacterial communities are influenced by variation in plant genetic background and use of different agricultural practices (Daqi et al., 2019; Deng et al., 2019; Feng et al., 2016; Lazcano et al., 2021; Mazzola et al., 2018). However, it is unknown if underlying ecological processes influencing soil bacterial communities are consistent across different strawberry production systems.

The goal of this study was to characterize soil bacterial communities in commercial strawberry production systems in a primary strawberry production region in California. Based on the highly uniform agronomic practices used to establish and maintain strawberry fields (Bolda et al., 2015), the primary hypothesis was that the influence of soil chemical characteristics, dispersal limitation, and deterministic versus stochastic community assembly processes would be consistent among different locations and plots. By focusing on bacterial communities in commercial strawberry production systems, this study provides novel insight into the ecology of bacteria in agricultural soil and informs future studies focused on their management to improve strawberry production.

## 2 MATERIALS AND METHODS

### 2.1 Field design and soil sampling

**FIGURE 1:**
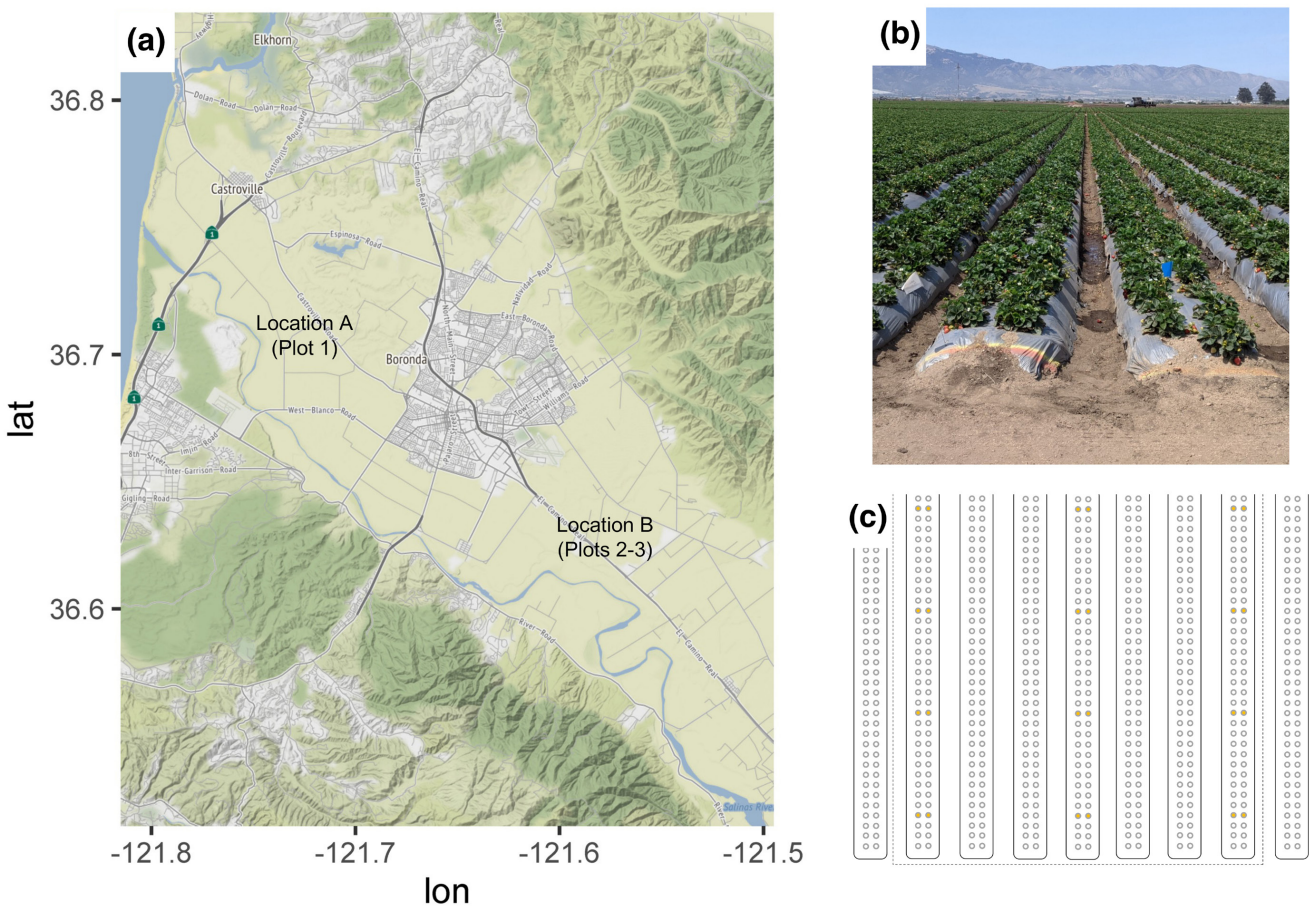
Overview of sampled commercial strawberry production locations and plots in the Salinas Valley, CA. Sub‐figure (a) is a map showing approximate locations of the two sampled strawberry production locations. Sub‐figure (b) is a photo of strawberry beds at one of the two sampled locations. Sub‐figure (c) is a cartoon showing sampling within individual plots. The plot is delimited by the dashed border. Each circle represents a strawberry plant and orange plants indicate location along a bed where soil samples were collected in the plot.

This study focused on two commercial strawberry production locations in the Salinas Valley region of California, USA. The sites were located northwest and southeast of the city of Salinas (Figure 1a). At both locations, fields were prepared in September 2021 with an initial pre‐plant broadcast application of the soil fumigant Triform 80 (TriCal Inc.). At Location A, fumigant was applied at a single regular rate (440 lbs/A). At location B, fumigant was applied at a reduced rate (330 lbs/A) and regular rate (440 lbs/A) in two separate areas of the field. Strawberry cv. Cabrillo were planted into two‐row beds in November 2021 and maintained using standard commercial strawberry production practices. Soil samples were collected on June 16th, 2021, about 8 months after planting and during peak strawberry production. Soil was sampled using a spatially explicit design from three plots. Plot 1 was at Location A and Plots 2–3 were at Location B. Plot 2 at Location B was established where the reduced fumigation rate was used and Plot 3 was established where the regular fumigation rate was used. Plot 2 and Plot 3 had the same orientation in the larger field and were separated from each other by six intervening strawberry beds (ca. 88 m or 290 ft). Each individual plot at both locations was composed of three strawberry beds, separated from each other by two beds, and covering ca. 122 m^2^ (1312 ft^2^; Figure 1b,c). Eight soil samples were collected from each bed, four from each side of the bed where strawberry plants were established. Each position sampled along the length of a bed was separated by nine plants (Figure 1c). Samples across a single bed at the same position along a bed were separated by ca. 56 cm (22 in). Each plant (including unsampled plants) along a bed were separated by 56 cm (22 in). Two 0.8 cm × 2 cm soil cores, separated by 2 cm were taken from the shoulder of the bed using a probe and pooled as a composite sample in plastic bags. Soil samples were stored at −20°C until further processing.

### 2.2 Soil DNA extraction and chemical characterization

Soil samples were passed through a 4000 μm sieve prior to DNA extraction to remove large stones and plant debris. Soil DNA was extracted using bead beating and the DNeasy PowerSoil Pro Kit (Qiagen). Soil samples were initially disrupted using 2, 30‐s pulses on a Mini‐BeadBeater‐16 (BioSpec Products) and eluted in a final volume of 50 μl. Extracted DNA was quantified using a NanoDrop ONE spectrophotometer (Thermo Fisher Scientific) and Qubit 3 fluorometer (Fisher Scientific). For soil chemical measurements, soil was passed through a 2000 μm sieve. Soil pH was measured in a 1:1 soil: distilled water suspension using a pH meter following published protocols (Thomas, 1996). Percent carbon and nitrogen of soil samples was measured at the University of Idaho Analytical Sciences Laboratory (https://www.uidaho.edu/cals/analytical‐sciences‐laboratory) using the combustion method with a CN628 Series Elemental Determinator (Leco).

### 2.3 16 S rRNA sequencing and classification

Bacterial community diversity and composition was characterized by sequencing the V3‐V4 hypervariable region of the 16 S rRNA locus using universal bacterial 16 S rRNA primers 341f (5′‐CCTACGGGAGGCAGCAG‐3′) and 806r (5′‐GGACTACHVGGGTWTCTAAT‐3′). Amplicon libraries were prepared and sequenced at the Michigan State University RTSF Genomics Core (https://rtsf.natsci.msu.edu/genomics/). Paired‐end sequencing was conducted using 500 cycles and a v2 reagent kit on the Illumina MiSeq platform (Illumina Inc.). Two negative control samples were included during library preparation and sequencing. Primer sequences were removed from demultiplexed sequence data using cutadapt v3.2. Sequence data were further processed to identify amplicon sequence variants (ASVs) using dada2 v1.6.0 implemented in R v3.4.1 (Callahan et al., 2016; R Development Core Team, 2015). Default values of the dada2 pipeline were used according to established protocols (https://benjjneb.github.io/dada2/bigdata_paired.html), except merged sequences below 400 bp were filtered prior to chimera identification and removal. Taxonomic classification of ASVs was conducted using The Ribosomal Database Project (RDP) Classifier implemented in mothur v.1.44.2 and the SILVA 132 database (Quast et al., 2013; Schloss et al., 2009; Wang et al., 2007). Sequences classified as chloroplast, mitochondria, or not assigned to a bacterial phylum were removed based on the assumption that they were not derived from bacteria. Following removal of singleton taxa (i.e., taxa represented by a single sequence in only one sample), sequence data were rarefied to the lowest sequence depth (9555 sequences). Operational taxonomic units (OTUs) were generated from ASV data by aligning sequence data with DECIPHER v2.22.0 and clustering at 97% identity using mothur v.1.44.2 (Schloss et al., 2009; Wright, 2016). Raw sequence data were archived at NCBI under BioProject PRJNA801672.

### 2.4 Statistics

Statistical analyses were conducted using R v4.1.2 (R Development Core Team, 2015). Initial analyses focused on variation in bacterial community diversity and composition among the total 72 samples collected across both locations and three plots. Analysis of variance (ANOVA) followed by TukeysHSD post‐hoc test was used to test for a plot effect on soil chemical characteristics, bacterial diversity, and bacterial richness. Data that deviated from a normal distribution based on quantile‐quantile (QQ) plots and Shapiro–Wilk's test were also evaluated using the non‐parametric Kruskal‐Wallis rank sum test. Significant differences among groups and corrections for multiple comparisons were determined using Dunn's test with the FSA v0.9.3 package (Ogle et al., 2022). Multiple regression was used to test for a significant correlation between soil chemical characteristics and bacterial diversity or richness. Principal coordinates analysis (PCoA) was used to evaluate and visualize variation in bacterial community composition using the package ade4 v1.7.18 (Dray & Dufour, 2007). Constrained ordination using redundancy analysis (rda) was used to test if soil chemical characteristics or location were significant predictors of bacterial community composition across all 72 samples. For rda tests, individual chemical characteristics were standardized using the z‐score transformation to account for differences in units (Ramette, 2007). For full models that were statistically significant (*p* < .05) and included all predictors, significance of individual predictors was calculated based on their effects while including all other predictors in the model (i.e., marginal effects).

Additional analyses focused on spatial structure and soil chemical characteristics as predictors of bacterial community composition within each of the three plots. Partial Mantel tests were used to test for a significant correlation between bacterial community composition using Bray–Curtis dissimilarity and spatial distance among samples, while accounting for variation in soil chemical characteristics. Significance of the Mantel statistic was assessed based on a null distribution generated using 999 permutations of the first distance matrix. Redundancy analysis was used to test the effect of soil chemical characteristics on bacterial community composition in each plot as described above. Mantel and rda tests were conducted for ASV and OTU data using the R package vegan v2.5.7 (Oksanen et al., 2020). The relationship between bacterial genera represented by more than 100 sequences and individual soil chemical characteristics within each plot was tested using DESeq v1.34.0 (Love et al., 2014).

A previously developed null‐modeling approach was used to measure the frequency of deterministic and stochastic community assembly processes impacting soil bacterial communities (Stegen et al., 2012, 2013, 2015). This approach compares pairwise estimates of phylogenetic and taxonomic turnover among local communities to a null distribution (reviewed in Zhou & Ning, 2017). In this study, local communities were defined based on plots. An underlying assumption of this null modeling approach is that closely related taxa display greater similarity in niches compared to distantly related taxa (Stegen et al., 2012; Stegen et al., 2013). To test this assumption, pairwise differences between taxa in niche and phylogenetic distances were calculated. Niche differences between bacterial taxa were calculated with the R package iCAMP v1.3.4 (Ning et al., 2020) using the taxon abundance‐weighted mean of individual soil chemical characteristics, as previously described (Stegen et al., 2012). Phylogenetic distances among taxa were calculated using the R package picante v1.8.2 (Kembel et al., 2010). A correlation between phylogenetic and niche distances was calculated using a Mantel test at multiple distance classes with the package vegan. The Beta‐Nearest Taxon Index (*β*‐NTI) was used to estimate significant deviations of a given community from a null distribution of the Beta‐Near Taxon Distance (*β*‐MNTD), where |*β*‐NTI| > 2 is evidence of deterministic processes and |*β*‐NTI| < 2 is evidence of stochastic processes (Stegen et al., 2012). Specifically, *β*‐NTI > 2 represents high levels of phylogenetic turnover among communities due to heterogenous (i.e., variable) selection. In contrast, *β*‐NTI < 2 indicates limited phylogenetic turnover among communities due to homogenous selection (Stegen et al., 2012, 2013). For pairwise communities with |*β*‐NTI| < 2, the Raup‐Crick Bray–Curtis index (RC_bray_) was calculated to classify different stochastic processes. Values of RC_bray_ < 0.95 indicate homogenizing dispersal and RC_bray_ > 0.95 dispersal limitation. Values of |RC_bray_| < 0.95 cannot be attributed to dispersal limitation or homogenizing dispersal, collectively referred to as *undominated* stochastic processes (Stegen et al., 2015; Zhou & Ning, 2017). To calculate these metrics and generate null distributions, phylogenetic trees were constructed from bacterial 16 S rRNA ASV and OTU data. Sequence data were initially aligned using DECIPHER as described above. A soft mask filter was applied to the alignment to remove columns with more than 50% missing data. Phylogenetic trees were constructed for data from each plot using FastTree2 v2.2.10 (Price et al., 2010). Pairwise estimates of *β*‐NTI and RC_bray_ for communities in each plot were calculated and null distributions were generated using the R package iCAMP v1.3.4 (Ning et al., 2020).

## 3 RESULTS

### 3.1 Bacterial communities across locations

Seventy‐two soil samples were analyzed from two strawberry production locations and three plots in the Salinas Valley, CA. The Plot 1 at Location A received regular rate of pre‐plant soil fumigation. At Location B, Plot 2 received a reduced fumigation rate, while Plot 3 received a regular fumigation rate. Based on Kruskal‐Wallis tests, there were significant differences in carbon, nitrogen, and pH among the three sampled plots. Percent carbon and nitrogen were significantly higher in Plot 1 at Location A compared to Plots 2 and 3 at Location B. In contrast, soil pH was significantly lower in Plot 1 compared to Plots 2 and 3 (Table 1).

**TABLE 1:** Summary of sampled plots and variation in soil chemical characteristics

Location (plot)	Carbon^a^	Nitrogen^b^	pH^c^
A (Plot 1)	2.042 ± 0.058a	0.213 ± 0.008a	6.454 ± 0.112a
B (Plot 2)	0.713 ± 0.078b	0.081 ± 0.011b	6.672 ± 0.190b
B (Plot 3)	0.721 ± 0.139b	0.084 ± 0.012b	6.651 ± 0.275b

^a^ Percent carbon of soil samples. Letters represent significant differences among groups based on the Kruskal‐Wallis non‐parametric test and Dunn's post‐hoc test.

^b^ Percent nitrogen of soil samples. Letters represent significant differences among groups based on the Kruskal‐Wallis non‐parametric test and Dunn's post‐hoc test.

^c^ pH of soil samples. Letters represent significant differences among groups based on the Kruskal‐Wallis non‐parametric test and Dunn's post‐hoc test.

**FIGURE 2:**
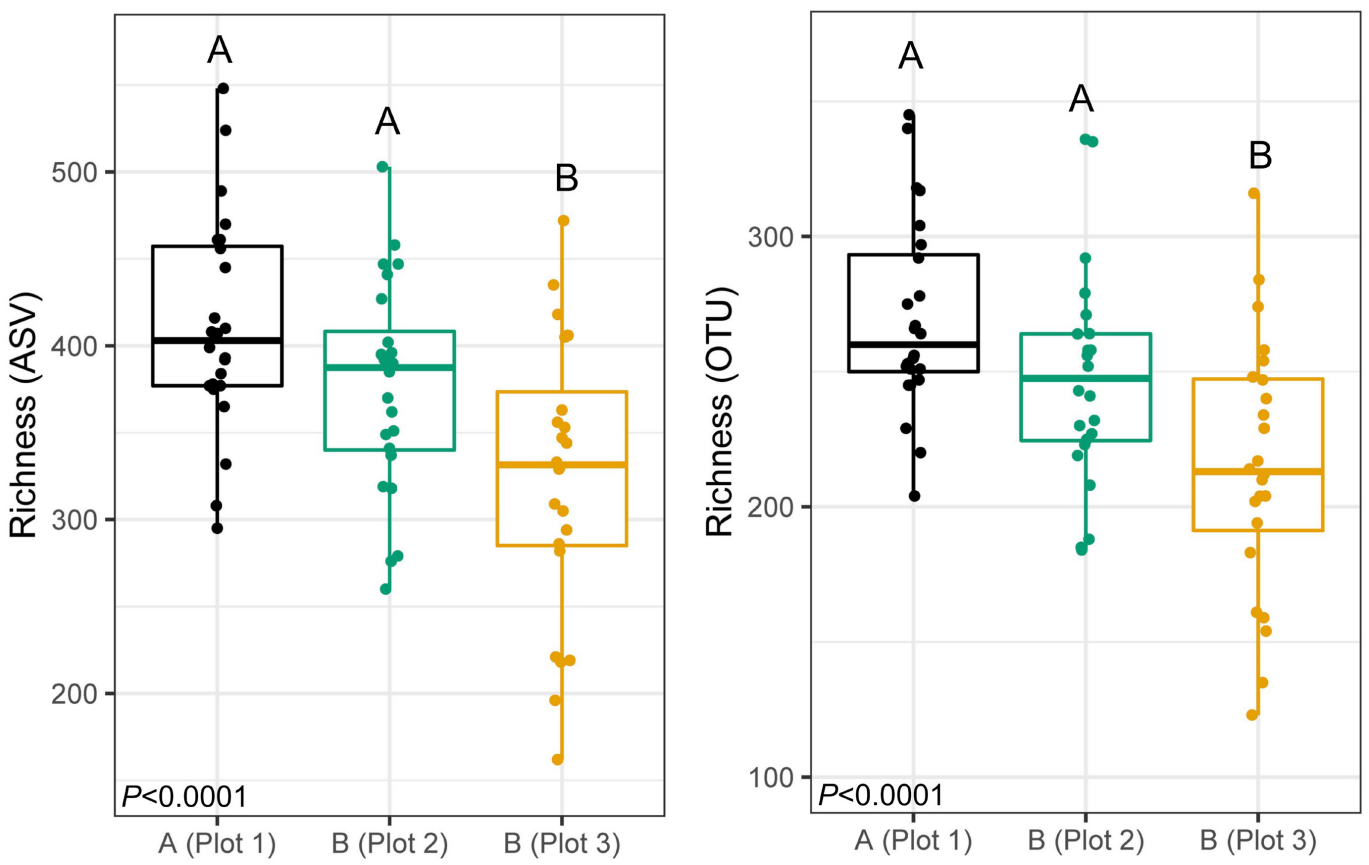
Bacterial richness (*y*‐axis) among sampled plots at location A and location B based on amplicon sequence variant (ASV) and operational taxonomic unit (OTU) data. Letters above boxplots represent groups that are significantly different from each other based on analysis of variance (ANOVA) followed by TukeyHSD post‐hoc tests.

A total of 7557 bacterial 16 S rRNA amplicon sequence variants (ASVs) were identified in the 72 soil samples, which clustered into 3030 operational taxonomic units (OTUs). The bacterial phylum with the greatest number of sequences was Proteobacteria, followed by Actinobacteria, and Chloroflexi. The most abundant genera among all samples were *Pseudoarthrobacter* (Microccaceae, Actinobacteria), followed by *Sphingomonas* (Sphingomonadaceae, Proteobacteria), and *Streptomyces* (Streptomycetaceae, Actinobacteria). Average bacterial richness was significantly different among the three sampled plots based on ASV (*F* = 9.809, *p* < .0001) and OTU (*F* = 10.71, *p* < .0001) data. Post‐hoc comparisons showed bacterial richness was significantly lower in Plot 3 compared to Plot 1 and Plot 2 (Figure 2). There was no significant difference in bacterial diversity among plots. Similarly, multiple regression showed individual soil chemical characteristics were not significantly correlated with bacterial richness or diversity (data not shown).

**FIGURE 3:**
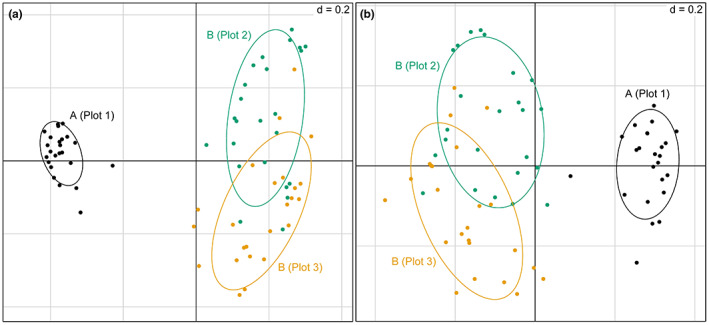
Ordination plots showing variation in bacterial community composition based on principal coordinates analysis among soil samples from two strawberry production systems and three plots. Samples from location (a and b) are labeled and followed by plot number in parentheses. Sub‐figure 9a) represents data classified as amplicon sequence variants (ASVs) and (b) as operational taxonomic units (OTUs).

Principal coordinates analysis showed a clear separation in ordination space of bacterial communities from Location A and B and an overlap of communities in Plot 2 and 3 at Location B. Similar relationships among bacterial communities among plots were observed using ASV and OTU data (Figure 3a,b). Despite having the same number of samples and same spatial distances among samples in each plot, ASV data also demonstrated greater variation in community composition within the two plots from Location B than the single plot at Location A (Figure 3a). Constrained ordination using redundancy analysis (rda) showed that the overall model testing the effect of location, soil carbon, soil nitrogen, and soil pH was significant for ASV (*F* = 4.0879, *p* < .001) and OTU (*F* = 4.0879, *p* < .001) data. Analysis of individual model terms showed location was a significant predictor of bacterial community composition across the 72 samples (*F*_ASV_ = 2.3054, *p* < .05; *F*_OTU_ = 3.3315, *p* < .05), but none of the soil chemical characteristics were statistically significant predictors within the same model. Together, these results show the composition of bacterial communities in the sampled strawberry production systems are differentiated by locations.

### 3.2 Bacterial communities within plots

**FIGURE 4:**
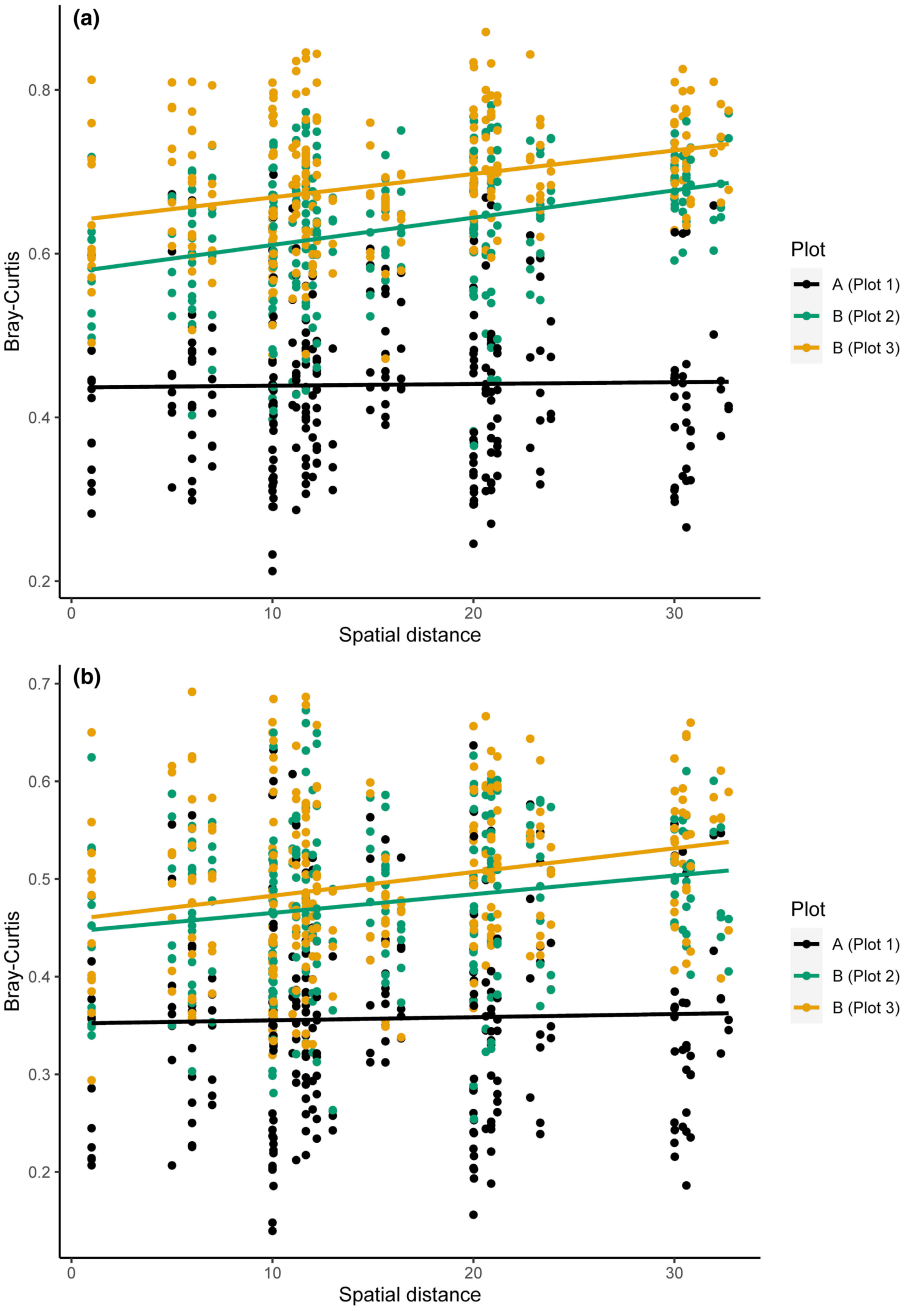
Scatter‐plots showing the relationship between bacterial community composition and spatial distance among samples from two strawberry production systems and three plots. Bray–Curtis dissimilarity index (*y*‐axis) represents compositional differences among samples and spatial distance (*x*‐axis) represents spatial distance among samples. Mantel tests show a significant (*p* < .05) correlation between community and spatial distance matrices in Plot 2 and Plot 3, but not Plot 1 (Table 2). Sub‐figure (a) represents data classified as amplicon sequence variants (ASVs) and (b) as operational taxonomic units (OTUs).

Subsequent analyses focused on bacterial communities within the three sampled plots at the two locations to determine if spatial location within strawberry production systems or soil chemical characteristics were consistent predictors of bacterial communities. As shown in Figure 4, Bray–Curtis distance among bacterial communities increased with increasing spatial distance among samples in Plot 2 (reduced fumigation rate) and Plot 3 (regular fumigation rate) at Location B based on ASV and OTU data. In contrast, Bray–Curtis distance did not clearly increase with increasing spatial distance in Plot 1 at Location A (Figure 2). Comparison of community dissimilarity and spatial distances, while accounting for differences in soil chemical characteristics among samples, showed a significant correlation between community composition and spatial distance in Plot 2 and Plot 3, but not Plot 1 (Table 2).

**TABLE 2:** Partial mantel tests evaluating the correlation between bacterial community composition and spatial distance among samples

Dataset	Location (plot)	Mantel‐statistic^a^
ASV	A (Plot 1)	0.0193
	B (Plot 2)	0.3221**
	B (Plot 3)	0.2995**
OTU	A (Plot 1)	0.02845
	B (Plot 2)	0.2054*
	B (Plot 3)	0.232**

*Note*: *p*‐values are represented as follows: * < .05;** < .01.

^a^ Mantel‐statistic from the partial Mantel test. Values represent the correlation between bacterial community composition (Bray–Curtis index) and spatial distance, while controlling for variation in soil chemical characteristics.

Constrained ordination showed soil chemical characteristics influenced bacterial community composition in one of the three sampled plots. In Plot 1 at Location A and Plot 3 at Location B where a regular fumigation rate was used, neither model was significant and none of the three chemical characteristics explained a significant amount of variation in bacterial community composition based on ASV or OTU data (Table 3). However, in Plot 2 at Location B where a reduced fumigation rate was used, the overall model was significant for ASV (*F* = 1.814, *p* < .05) and OTU (*F* = 2.8347, *p* < .01) data. As shown in Table 3, bacterial community composition was significantly influenced by soil pH and nitrogen in this plot. Multiple regression indicated bacterial richness and diversity within the three plots were not significantly correlated with soil carbon, nitrogen, or pH (data not shown). Further analysis within plots showed soil carbon in Plot 2 at Location B was a significant predictor of four bacterial genera. An unclassified genus in the family Bacillaceae showed a significant positive association with soil carbon. In contrast, an unclassified genus in the family Rhodobacteraceae as well as the genera *Novosphingobium* and *Porphyrobacter* showed a negative association with soil carbon.

**TABLE 3:** Constrained ordination testing the effect of soil chemical characteristics on soil bacterial community composition within plots

Dataset^a^	Location (plot)	Predictor^b^	*F*‐statistic^c^
ASV	A (Plot 1)	carbon	0.7964
		nitrogen	0.4709
		pH	0.9983
	B (Plot 2)	carbon	1.6795
		nitrogen	2.1588*
		pH	2.1197*
	B (Plot 3)	carbon	0.3925
		nitrogen	1.1403
		pH	0.9158
OTU	A (Plot 1)	carbon	0.5408
		nitrogen	0.4277
		pH	1.1458
	B (Plot 2)	carbon	1.951
		nitrogen	3.5585*
		pH	3.1607*
	B (Plot 3)	carbon	0.1791
		nitrogen	0.5056
		pH	0.5692

*Note*: *p*‐values are represented as follows: * < .05;** < .01.

^a^ Data used in the analysis. ASV, amplicon sequence variants; OTU, operational taxonomic units.

^b^ Predictors included in the redundancy analysis (rda) constrained ordination model.

^c^ *F*‐statistic output from the rda model. Values represent marginal effects of each predictor based on individual contributions to model when all other predictors are included.

### 3.3 Community assembly processes within plots

**FIGURE 5:**
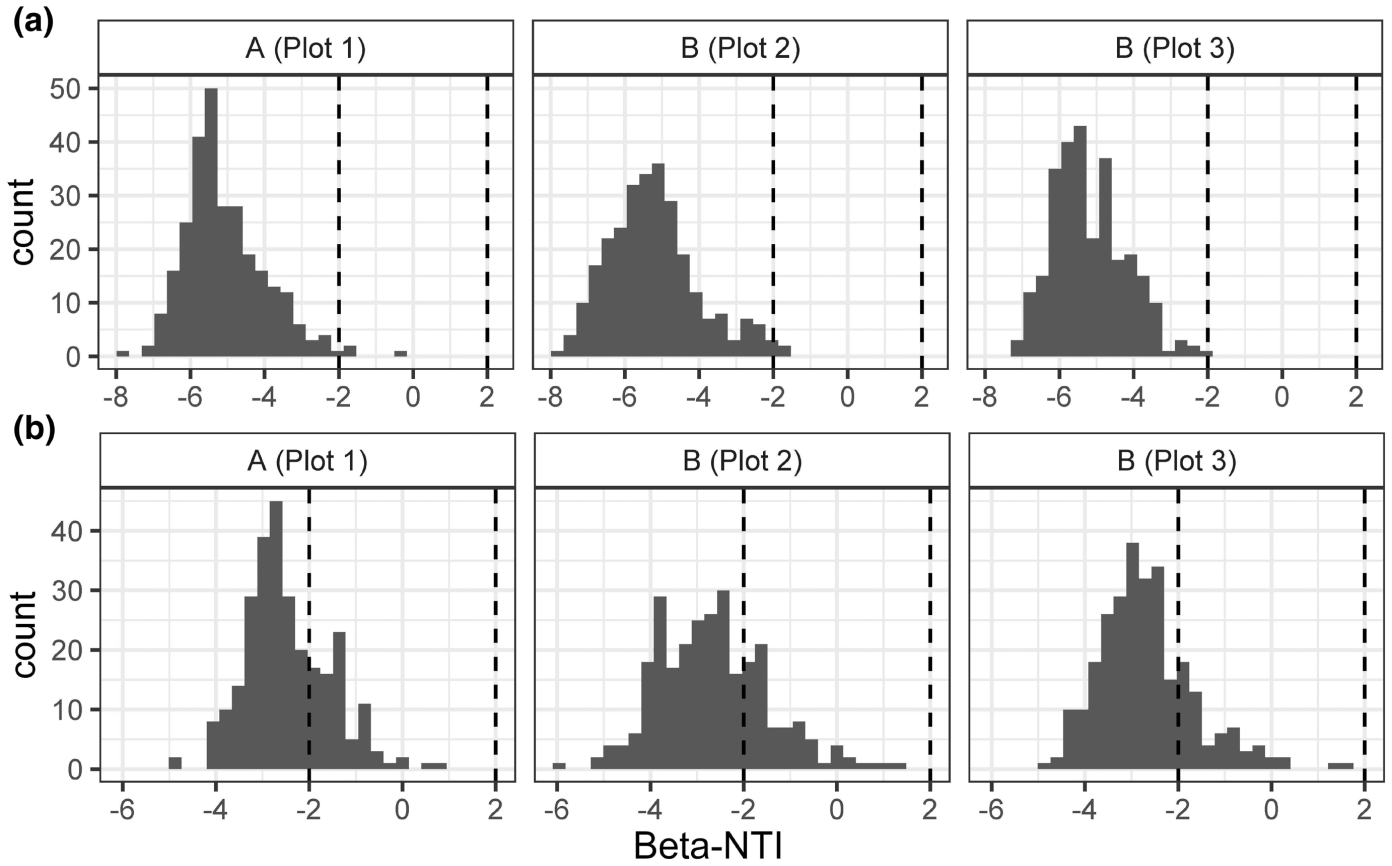
Histograms showing the Beta‐Nearest Taxon Index (*β*‐NTI) distributions for pairwise comparisons among bacterial communities within sampled plots. Sub‐figure (a) shows data for amplicon sequence variant (ASV) data and sub‐figure (b) shows operational taxonomic unit (OTU) data. Dashed vertical lines represent primary designation for whether community assembly processes are classified as deterministic |*β*‐NTI| > 2 or stochastic |*β*‐NTI| < 2.

Bacterial communities within each plot showed a significant positive correlation between niche distances (i.e., the chemical characteristic values where a taxon was most abundant) and phylogenetic distances for ASV and OTU data. Most of the significant positive correlations between niche and phylogenetic distances were among the smallest phylogenetic distance classes (i.e., closest related taxa; Tables A1–A6). Calculation of *β*‐NTI values among communities showed a reduced amount of phylogenetic turnover within each plot with most estimates lower than −2 (Figure 5). As shown in Figure 5 and Table 4, the distribution of *β*‐NTI values for OTU data were consistently higher than ASV data. Based on a *β*‐NTI value cutoff of −2, more than 99% of pairwise comparisons in ASV data were classified as the deterministic process of homogenous selection as the primary factor influencing bacterial communities in each of the three plots. Additional ASV data were classified as stochastic processes of dispersal limitation (0.1%) and unclassified stochastic processes not attributed to dispersal (i.e., undominated; 0.5%). In contrast, OTU data showed 74% of these data were classified as homogenous selection and 5% as dispersal limitation. The remaining 21% were classified as stochastic processes not attributed to dispersal. Operational taxonomic unit data showed evidence of dispersal limitation was more frequent in Plot 2 (7%) and Plot 3 (8%) than Plot 1 (0.7%). Together, these data suggest the deterministic process of homogenous selection is the primary community assembly process, but stochastic processes are also influencing bacterial communities in the sampled plots.

**TABLE 4:** Null model classification of community assembly processes in commercial strawberry production systems

Dataset^a^	Location (plot)	Variable selection^b^	Homogenous selection^c^	Dispersal limitation^d^	Homogenizing dispersal^e^	Undominated^f^
ASV	A (Plot 1)	0	273	0	0	3
	B (Plot 2)	0	274	1	0	1
	B (Plot 3)	0	276	0	0	0
	Total	0	823	1	0	4
OTU	A (Plot 1)	0	197	2	0	77
	B (Plot 2)	0	201	19	0	56
	B (Plot 3)	0	218	21	0	37
	Total	0	616	42	0	170

^a^ Data used in the analysis. ASV, amplicon sequence variants; OTU, operational taxonomic units (0.97 similarity).

^b^ The deterministic process of variable selection is based on Beta‐Nearest Taxon Index (*β*‐NTI) > 2.

^c^ The deterministic process of homogenous selection is based on *β*‐NTI < ‐2.

^d^ The stochastic process of dispersal limitation is based on Raup‐Crick Bray–Curtis index (RC_bray_) < 0.95.

^e^ The stochastic process of homogenizing dispersal is based on RC_bray_ > 0.95.

^f^ The stochastic processes that cannot be attributed to dispersal (i.e., undominated) are based on |RC_bray_| < 0.95.

## 4 DISCUSSION

Despite the importance of soil bacterial communities to crop health, relatively little research has focused on ecological processes influencing bacterial communities in commercial strawberry production systems. This study applied spatially explicit soil sampling to characterize bacterial communities within two strawberry production locations in the Salinas Valley region of California. Bacterial community composition was differentiated between the two production locations. There was evidence of spatial structure and dispersal limitation within plots only at one location. Soil nitrogen and pH were significant predictors of bacterial community composition in one of two neighboring plots at the same location. Within all plots, there was a high frequency of reduced phylogenetic turnover among communities suggesting homogenous selective pressures played a primary role in structuring bacterial communities. Overall, these results demonstrate differences in the composition and ecological drivers of bacterial communities between different strawberry production sites in the same geographic region.

The composition of soil bacterial communities was clearly differentiated between the two strawberry production locations which were separated by more than 10 miles from each other in the Salinas Valley. Prior studies have also found geographic location is a primary factor influencing bacterial community composition in soil of other specialty crop production systems. For example, soil bacterial communities sampled at multiple lettuce production sites in the Salinas Valley were differentiated from lettuce fields in southern California and Arizona (Ma et al., 2016). Similarly, the composition of prokaryotic and eukaryotic microbial communities in soil of apple orchards was influenced by different locations or production sites (Bintarti et al., 2020; Deakin et al., 2018). The differentiation in bacterial communities observed in this study could be a result differing soil characteristics or land use histories between the two locations. Soil carbon, nitrogen, and pH were significantly different between the two locations. However, these chemical characteristics did not explain a significant amount of variation in bacterial community composition across the 72 soil samples. Limited or an absence of bacterial dispersal between the two locations could also have contributed to this differentiation in community composition.

There was inconsistent evidence of spatial structure and dispersal limitation of bacterial communities among the three plots sampled in this study. In particular, communities in the two plots at Location B, but not within the single plot at Location A, showed a significant increase in dissimilarity among communities with increasing spatial distance. Similar distance‐decay patterns of microbial communities have been well‐documented in soil across multiple spatial scales (Burns et al., 2015; Clark et al., 2021; Durrer et al., 2017). In this study, the distance‐decay pattern was statistically significant after accounting for variation in soil carbon, nitrogen, and pH. This pattern could be a result of dispersal limitation within the sampled 122 m^2^ area plots or the effect of an unmeasured variable that displays spatial structure (Hanson et al., 2012). Interestingly, Location A where no distance‐decay pattern was observed, was previously used to grow vegetable crops, while Location B has a history of strawberry production. Because vegetable crops have much shorter production cycles, there would likely be much greater frequency of tillage over the same time‐span at Location A compared to Location B. This potential increased tillage frequency and different land‐use history could explain the absence of a distance‐decay pattern at Location A (Navarro‐Noya et al., 2013; Rodrigues et al., 2013; Wang, Liu, et al., 2022).

The influence of soil nitrogen and pH on bacterial community composition observed in this study is consistent with prior studies focused on agricultural soils in California, including lettuce production systems in the Salinas Valley (Burns et al., 2015; Ma et al., 2016). However, the influence of these soil chemical characteristics was observed in only one of two neighboring plots separated by about 90 meters from each other in the same field. The primary known difference between these two plots was the level of soil fumigation applied prior to establishing the strawberry beds. Plot 2 where the influence of soil nitrogen and pH was observed received a reduced fumigation rate, whereas Plot 3 received the standard fumigation rate. Though this study is observational in nature, other replicated microcosm and field experiments have shown that soil fumigation alters the type and quantity of nitrogen present in soil as well as the diversity and composition of soil bacterial communities (Fang et al., 2020; Klose et al., 2006; Li et al., 2017; Yan et al., 2013). This could potentially mask the relationship between soil chemical characteristics and soil bacterial communities in the two plots that received regular fumigation rates. Future research will need to evaluate if the differential influence of soil chemical characteristics observed in the two plots of this study is due to variation in fumigant application rate.

The primary community assembly process observed in each plot was homogenous selection. This deterministic process accounted for the 99% of comparisons between samples for ASV data and 74% for OTU data. This was reflected in reduced levels of phylogenetic turnover among samples and suggests bacterial taxa in these communities were exposed to a uniform selective pressure. In the context of standard strawberry production practices, such uniform selective pressures could be from tillage, soil fumigation, or application of plastic tarps throughout the growing season (Bolda et al., 2015). Similar predominance of homogenous selection has also been observed in aquatic and terrestrial ecosystems, including agricultural soils (Allen et al., 2020; Barnett et al., 2019; Fodelianakis et al., 2021; Jiao & Lu, 2020; Wang, Feng, et al., 2022). Interestingly, less than 1% of comparisons among samples within plots were classified as stochastic processes for ASV data, while about 25% were classified as stochastic processes for OTU data. These results share similarities with a recent study suggesting the influence of deterministic processes on bacterial communities is more important at finer phylogenetic resolutions (Quiroga et al., 2022). Based on OTU data, communities from the two plots at Location B showed a greater frequency of dispersal limitation, compared to the plot at Location A. This provides a second line of evidence that bacterial communities that displayed distance‐decay relationships at Location B are partially influenced by dispersal limitation.

This observational study provides new insight into the ecology of soil bacterial communities in commercial strawberry production systems. It demonstrates that the influence of soil chemical characteristics and dispersal limitation on soil bacterial communities are not consistent among different locations and plots in the same production region. These results may explain why the efficacy of agricultural practices that rely on altering microbial activity in soil (e.g., use of organic soil amendments) are highly variable across different California strawberry production systems (Mazzola et al., 2018). Despite these novel results, the study is limited due to its observational nature and focus on a single production region. Future research should address similar questions under replicated experiments and other production regions in California, the US, and other countries. Ultimately, long‐term research efforts focused on managing the soil microbiome to improve strawberry production need to be based on a better understanding of underlying ecological processes that influence microbial taxa that improve soil and crop health.

## CONFLICT OF INTEREST

The authors have no conflict of interest.

## Supporting information


Table A1.

## Data Availability

Data are publicly accessible at NCBI under BioProject PRJNA801672.
