# Supplementary material for: Soil bacterial communities are influenced by soil chemical characteristics and dispersal limitation in commercial strawberry production systems
Source: Plant Environ Interact. 2023 Jan 11;4(1):11–22. doi: 10.1002/pei3.10099 (PMC10168042; doi:10.1002/pei3.10099)
Supplement: Supplementary file 1 — Table A1. [file PEI3-4-11-s001.docx]

Table A1. Correlation between phylogenetic and niche distance (carbon) for amplicon sequence variant (ASV) data.

| Location A (Plot 1) | | | Location B (Plot 2) | | | Location B (Plot 3) | | |
| --- | --- | --- | --- | --- | --- | --- | --- | --- |
| index^a^ | Mantel | P^c^ | index^a^ | Mantel | P^c^ | index^a^ | Mantel | P^c^ |
| 0.024 | 0.009 | 0.001 | 0.024 | 0.010 | 0.001 | 0.023 | 0.009 | 0.001 |
| 0.072 | 0.011 | 0.002 | 0.073 | 0.005 | 0.042 | 0.069 | 0.008 | 0.008 |
| 0.120 | 0.011 | 0.003 | 0.121 | 0.002 | 0.310 | 0.115 | 0.019 | 0.003 |
| 0.168 | 0.007 | 0.005 | 0.170 | 0.001 | 0.620 | 0.161 | 0.012 | 0.035 |
| 0.216 | 0.004 | 0.156 | 0.219 | -0.003 | 0.666 | 0.207 | 0.013 | 0.018 |
| 0.263 | 0.011 | 0.044 | 0.267 | -0.014 | 0.020 | 0.253 | 0.022 | 0.006 |
| 0.311 | -0.004 | 0.312 | 0.316 | -0.018 | 0.007 | 0.299 | 0.024 | 0.007 |
| 0.359 | 0.000 | 0.468 | 0.364 | -0.016 | 0.008 | 0.345 | 0.011 | 0.024 |
| 0.407 | -0.005 | 0.212 | 0.413 | -0.008 | 0.110 | 0.391 | -0.018 | 0.009 |
| 0.455 | -0.010 | 0.036 | 0.461 | -0.001 | 0.930 | 0.437 | -0.035 | 0.010 |
| 0.503 | -0.006 | 0.292 | 0.510 | 0.011 | 0.016 | 0.483 | -0.020 | 0.011 |
| 0.551 | -0.001 | 0.780 | 0.558 | 0.019 | 0.012 | 0.529 | -0.014 | 0.030 |
| 0.599 | 0.001 | 1.000 | 0.607 | 0.018 | 0.013 | 0.575 | -0.015 | 0.024 |
| 0.647 | -0.002 | 1.000 | 0.656 | 0.013 | 0.024 | 0.621 | -0.010 | 0.070 |

a. Distance class for which Mantel statistic was calculated.

b. Mantel test statistic.

c. P-value corrected for multiple comparisons using the methods described in Holm (1979).

Table A2. Correlation between phylogenetic and niche distance (carbon) for operational taxonomic unit (OTU) data.

| Location A (Plot 1) | | | Location B (Plot 2) | | | Location B (Plot 3) | | |
| --- | --- | --- | --- | --- | --- | --- | --- | --- |
| index^a^ | Mantel | P^c^ | index^a^ | Mantel | P^c^ | index^a^ | Mantel | P^c^ |
| 0.027 | 0.008 | 0.001 | 0.026 | 0.009 | 0.001 | 0.028 | 0.009 | 0.001 |
| 0.075 | 0.007 | 0.007 | 0.073 | 0.003 | 0.265 | 0.080 | 0.009 | 0.033 |
| 0.123 | 0.003 | 0.186 | 0.119 | 0.002 | 0.530 | 0.131 | 0.010 | 0.118 |
| 0.171 | 0.002 | 0.372 | 0.166 | -0.003 | 0.795 | 0.182 | 0.020 | 0.033 |
| 0.219 | 0.005 | 0.558 | 0.212 | -0.017 | 0.072 | 0.234 | 0.031 | 0.005 |
| 0.267 | 0.001 | 0.744 | 0.259 | -0.021 | 0.006 | 0.285 | 0.043 | 0.006 |
| 0.315 | -0.004 | 0.930 | 0.306 | -0.015 | 0.065 | 0.336 | 0.032 | 0.007 |
| 0.363 | -0.007 | 0.438 | 0.352 | -0.019 | 0.012 | 0.388 | -0.006 | 0.236 |
| 0.411 | -0.003 | 1.000 | 0.399 | -0.011 | 0.136 | 0.439 | -0.043 | 0.009 |
| 0.459 | 0.000 | 1.000 | 0.445 | 0.006 | 0.540 | 0.490 | -0.033 | 0.01 |
| 0.507 | 0.009 | 0.432 | 0.492 | 0.016 | 0.011 | 0.542 | NA | NA |
| 0.555 | 0.004 | 1.000 | 0.539 | 0.019 | 0.012 | 0.593 | -0.012 | NA |
| 0.603 | -0.003 | 1.000 | 0.585 | 0.016 | 0.018 | 0.644 | NA | NA |

a. Distance class for which Mantel statistic was calculated.

b. Mantel test statistic.

c. P-value corrected for multiple comparisons using the methods described in Holm (1979).

Table A3. Correlation between phylogenetic and niche distance (nitrogen) for amplicon sequence variant (ASV) data.

| Location A (Plot 1) | | | Location B (Plot 2) | | | Location B (Plot 3) | | |
| --- | --- | --- | --- | --- | --- | --- | --- | --- |
| index^a^ | Mantel | P^c^ | index^a^ | Mantel | P^c^ | index^a^ | Mantel | P^c^ |
| 0.024 | 0.010 | 0.001 | 0.024 | 0.014 | 0.001 | 0.023 | 0.007 | 0.001 |
| 0.072 | 0.012 | 0.002 | 0.073 | 0.009 | 0.002 | 0.069 | 0.008 | 0.008 |
| 0.120 | 0.009 | 0.008 | 0.121 | 0.010 | 0.003 | 0.115 | 0.011 | 0.016 |
| 0.168 | 0.003 | 0.201 | 0.170 | 0.011 | 0.004 | 0.161 | 0.012 | 0.024 |
| 0.216 | 0.005 | 0.248 | 0.219 | 0.004 | 0.090 | 0.207 | 0.007 | 0.074 |
| 0.263 | 0.016 | 0.008 | 0.267 | -0.012 | 0.006 | 0.253 | 0.008 | 0.088 |
| 0.311 | 0.015 | 0.010 | 0.316 | -0.014 | 0.007 | 0.299 | 0.005 | 0.148 |
| 0.359 | 0.013 | 0.012 | 0.364 | -0.013 | 0.008 | 0.345 | 0.000 | 0.482 |
| 0.407 | -0.001 | 0.432 | 0.413 | -0.006 | 0.104 | 0.391 | -0.009 | 0.145 |
| 0.455 | -0.013 | 0.016 | 0.461 | -0.001 | 0.312 | 0.437 | -0.008 | 0.210 |
| 0.503 | -0.019 | 0.011 | 0.510 | 0.004 | 0.270 | 0.483 | -0.006 | 0.296 |
| 0.551 | -0.020 | 0.012 | 0.558 | 0.008 | 0.050 | 0.529 | -0.010 | 0.153 |
| 0.599 | -0.016 | 0.020 | 0.607 | 0.013 | 0.013 | 0.575 | -0.011 | 0.130 |
| 0.647 | -0.013 | 0.025 | 0.656 | 0.010 | 0.042 | 0.621 | -0.006 | 0.384 |

a. Distance class for which Mantel statistic was calculated.

b. Mantel test statistic.

c. P-value corrected for multiple comparisons using the methods described in Holm (1979).

Table A4. Correlation between phylogenetic and niche distance (nitrogen) for operational taxonomic unit (OTU) data.

| Location A (Plot 1) | | | Location B (Plot 2) | | | Location B (Plot 3) | | |
| --- | --- | --- | --- | --- | --- | --- | --- | --- |
| index^a^ | Mantel | P^c^ | index^a^ | Mantel | P^c^ | index^a^ | Mantel | P^c^ |
| 0.027 | 0.008 | 0.001 | 0.026 | 0.012 | 0.001 | 0.028 | 0.008 | 0.001 |
| 0.075 | 0.009 | 0.003 | 0.073 | 0.011 | 0.003 | 0.080 | 0.010 | 0.016 |
| 0.123 | 0.013 | 0.003 | 0.119 | 0.013 | 0.004 | 0.131 | 0.011 | 0.074 |
| 0.171 | -0.002 | 0.366 | 0.166 | 0.005 | 0.132 | 0.182 | 0.012 | 0.118 |
| 0.219 | 0.004 | 0.576 | 0.212 | -0.008 | 0.212 | 0.234 | 0.001 | 0.467 |
| 0.267 | 0.016 | 0.075 | 0.259 | -0.012 | 0.048 | 0.285 | 0.013 | 0.236 |
| 0.315 | 0.009 | 0.285 | 0.306 | -0.003 | 0.320 | 0.336 | 0.010 | 0.295 |
| 0.363 | 0.003 | 0.864 | 0.352 | -0.010 | 0.084 | 0.388 | 0.000 | 0.934 |
| 0.411 | 0.003 | 1.000 | 0.399 | -0.012 | 0.054 | 0.439 | 0.000 | 1.000 |
| 0.459 | -0.004 | 1.000 | 0.445 | 0.001 | 0.640 | 0.490 | -0.008 | 0.592 |
| 0.507 | -0.009 | 0.511 | 0.492 | 0.011 | 0.040 | 0.542 | NA | NA |
| 0.555 | -0.013 | 0.416 | 0.539 | 0.010 | 0.084 | 0.593 | -0.014 | NA |
| 0.603 | -0.017 | 0.160 | 0.585 | 0.005 | 0.530 | 0.644 | NA | NA |

a. Distance class for which Mantel statistic was calculated.

b. Mantel test statistic.

c. P-value corrected for multiple comparisons using the methods described in Holm (1979).

Table A5. Correlation between phylogenetic and niche distance (pH) for amplicon sequence variant (ASV) data.

| Location A (Plot 1) | | | Location B (Plot 2) | | | Location B (Plot 3) | | |
| --- | --- | --- | --- | --- | --- | --- | --- | --- |
| index^a^ | Mantel | P^c^ | index^a^ | Mantel | P^c^ | index^a^ | Mantel | P^c^ |
| 0.024 | 0.012 | 0.001 | 0.024 | 0.010 | 0.001 | 0.023 | 0.007 | 0.001 |
| 0.072 | 0.017 | 0.002 | 0.073 | 0.010 | 0.002 | 0.069 | 0.007 | 0.002 |
| 0.120 | 0.017 | 0.003 | 0.121 | 0.014 | 0.003 | 0.115 | 0.009 | 0.010 |
| 0.168 | 0.007 | 0.017 | 0.170 | 0.008 | 0.012 | 0.161 | 0.003 | 0.226 |
| 0.216 | 0.006 | 0.079 | 0.219 | 0.000 | 0.477 | 0.207 | 0.004 | 0.388 |
| 0.263 | 0.020 | 0.006 | 0.267 | 0.000 | 0.912 | 0.253 | 0.016 | 0.008 |
| 0.311 | -0.008 | 0.106 | 0.316 | 0.012 | 0.028 | 0.299 | 0.012 | 0.016 |
| 0.359 | -0.010 | 0.032 | 0.364 | 0.016 | 0.008 | 0.345 | -0.001 | 0.582 |
| 0.407 | -0.013 | 0.010 | 0.413 | 0.007 | 0.099 | 0.391 | -0.014 | 0.009 |
| 0.455 | -0.012 | 0.025 | 0.461 | -0.002 | 0.855 | 0.437 | -0.014 | 0.010 |
| 0.503 | -0.001 | 0.448 | 0.510 | -0.007 | 0.125 | 0.483 | -0.004 | 0.668 |
| 0.551 | 0.006 | 0.266 | 0.558 | -0.012 | 0.012 | 0.529 | -0.004 | 0.835 |
| 0.599 | 0.003 | 0.502 | 0.607 | -0.018 | 0.013 | 0.575 | -0.008 | 0.186 |
| 0.647 | -0.001 | 0.896 | 0.656 | -0.015 | 0.014 | 0.621 | -0.007 | 0.372 |

a. Distance class for which Mantel statistic was calculated.

b. Mantel test statistic.

c. P-value corrected for multiple comparisons using the methods described in Holm (1979).

Table A6. Correlation between phylogenetic and niche distance (pH) for operational taxonomic unit (OTU) data.

| Location A (Plot 1) | | | Location B (Plot 2) | | | Location B (Plot 3) | | |
| --- | --- | --- | --- | --- | --- | --- | --- | --- |
| index^a^ | Mantel | P^c^ | index^a^ | Mantel | P^c^ | index^a^ | Mantel | P^c^ |
| 0.027 | 0.011 | 0.001 | 0.026 | 0.014 | 0.001 | 0.028 | 0.008 | 0.001 |
| 0.075 | 0.011 | 0.002 | 0.073 | 0.021 | 0.002 | 0.080 | 0.015 | 0.002 |
| 0.123 | 0.010 | 0.005 | 0.119 | 0.017 | 0.003 | 0.131 | 0.016 | 0.008 |
| 0.171 | 0.007 | 0.101 | 0.166 | 0.005 | 0.210 | 0.182 | 0.013 | 0.028 |
| 0.219 | 0.010 | 0.202 | 0.213 | -0.001 | 0.446 | 0.234 | 0.016 | 0.038 |
| 0.267 | -0.003 | 0.345 | 0.259 | 0.001 | 0.844 | 0.285 | 0.021 | 0.006 |
| 0.315 | -0.008 | 0.404 | 0.306 | 0.010 | 0.236 | 0.336 | 0.009 | 0.072 |
| 0.363 | -0.008 | 0.385 | 0.352 | 0.005 | 0.728 | 0.388 | -0.014 | 0.044 |
| 0.411 | -0.006 | 0.505 | 0.399 | 0.004 | 0.905 | 0.439 | -0.015 | 0.048 |
| 0.459 | -0.003 | 0.606 | 0.445 | 0.003 | 1.000 | 0.490 | -0.010 | 0.132 |
| 0.507 | 0.009 | 0.616 | 0.492 | -0.005 | 1.000 | 0.542 | NA | NA |
| 0.555 | 0.002 | 0.900 | 0.539 | -0.008 | 0.513 | 0.593 | -0.012 | NA |
| 0.603 | 0.003 | 1.000 | 0.585 | -0.007 | 0.728 | 0.644 | NA | NA |

a. Distance class for which Mantel statistic was calculated.

b. Mantel test statistic.

c. P-value corrected for multiple comparisons using the methods described in Holm (1979).
